# Cancer mortality projections through 2026 among young adults in 15 upper-middle and high-income countries with focus on colorectal cancer

**DOI:** 10.1007/s10552-026-02149-w

**Published:** 2026-04-03

**Authors:** Silvia Mignozzi, Claudia Santucci, Gianfranco Alicandro, Margherita Pizzato, Prabhat Jha, Eva Negri, Carlo La Vecchia

**Affiliations:** 1 Department of Clinical Sciences and Community Health, Department of Excellence 2023-2027, University of Milan, Via Giovanni Celoria 22, 20133 Milan, Italy; 2 Fondazione IRCCS Ca’ Granda Ospedale Maggiore Policlinico, Milan, Italy; 3 Department of Pathophysiology and Transplantation, University of Milan, Milan, Italy; 4 Mother and Child Department, Cystic Fibrosis Centre, Fondazione IRCCS Ca’ Granda Ospedale Maggiore Policlinico, Milan, Italy; 5 Centre for Global Health Research, Unity Health Toronto, University of Toronto, Toronto, ON Canada; 6 Nuffield Department of Population Health, University of Oxford, Oxford, UK; 7 Department of Medical and Surgical Sciences, University of Bologna, Bologna, Italy

**Keywords:** Early-onset cancer, Cancer mortality, Colorectal cancer, Projections

## Abstract

**Background:**

An increasing cancer incidence has been observed among recent generations over the last decade. However, apart from colorectal cancer, these increases were not associated with rises in mortality. This study aimed to project early-onset cancer mortality trends through 2026.

**Methods:**

We extracted death counts for individuals aged 25–49 from the WHO Mortality Database and population data from the UN World Population Prospects. The analysis included the 15 most populous upper-middle and high-income countries with valid mortality data from 1990 to 2023, plus the EU-27. Projections of the number of deaths and age-standardized mortality rates (ASMRs) for 2026 were obtained using Poisson regression models fitted to death counts, with the logarithm of population as the offset term. The models were fitted on data from the most recent trend segment identified through joinpoint regression.

**Results:**

In the EU-27, ASMRs from all cancers combined are projected to decrease by 2026 (males: -17.2% as compared to 2019–2021; females: -13.4%). Declines are expected in all countries for all cancers combined and lung cancer, and for 13/15 countries for breast cancer. Colorectal cancer mortality is projected to increase substantially in the UK (males: + 26.9%; females: + 21.2%), and to smaller extents in the USA, selected Latin American countries and Australian females. Pancreatic cancer mortality showed heterogeneous trends across countries and sexes.

**Conclusions:**

Overall, cancer mortality at age 25–49 years is projected to decline by 2026. Colorectal cancer mortality is expected to increase in the coming years in some countries, possibly reflecting the increased prevalence of overweight, obesity, and related metabolic conditions, including diabetes.

**Supplementary Information:**

The online version contains supplementary material available at 10.1007/s10552-026-02149-w.

## Introduction

A rise in the incidence of early-onset cancer has been reported [1, 2], with upward trends observed for many cancer sites [3]. Colorectal cancer incidence is increasing more rapidly than that of other cancer sites, particularly in the USA, Canada, Australia and the UK [4, 5].

We recently reported that the rising incidence of colorectal cancer among young adults is accompanied by increasing mortality in some upper-middle- and high-income countries [6], likely reflecting the heterogeneity in the growing prevalence of overweight and obesity across different areas of the world.

In this study, we further investigate the implications of these recent trends in cancer mortality among young adults by providing projections through 2026 for the 15 most populous high- and middle-income countries worldwide, as well as for the EU-27. By focusing on short-term projections, this analysis offers timely data to support public health planning in cancer prevention. The study also provides age-specific evaluations of colorectal cancer mortality among individuals aged 30–39 and 40–49 years to identify which age groups are most affected by recent trends.

## Materials and methods

We retrieved death counts at ages 25–49 from 1990 up to 2023 (or the most recent available year) from the World Health Organization (WHO) mortality database [7] for all cancers combined and for the most common cancer sites, including colorectum, lung, pancreas, and breast. Countries classified by the World Bank [8] as high- or upper-middle-income were included if they had populations ≥ 15 million inhabitants, high data quality [9], defined according to completeness and usability, and recorded at least 500 annual cancer deaths among young adults aged 25–49 during the last triennium. Countries with civil registration coverage of the cause of death of less than 90% were excluded. From Europe, we included France, Germany, Italy, the Netherlands, Poland, Spain, and the UK, plus the EU-27; from the Americas, we included Canada, the USA, Mexico, Argentina, and Brazil; and from Australasia, Japan, the Republic of Korea and Australia. We retrieved mid-year resident population data and population projections from the United Nations World Population Prospects database [10].

Age-standardised mortality rates (ASMR) were derived using the world standard population [11, 12]. We compared ASMRs for the triennium 2019–2021 with those for 2009–2011 and computed the percentage change between the two periods. We fitted a joinpoint regression model [13, 14] to the ASMRs, over the period 1990 until 2023 or the most recent available year. We thus identified the joinpoint(s), that is, the year(s) when a change in the linear slope (on a log scale) of the temporal trend occurred, by testing up to a maximum of five joinpoints. The joinpoint analysis was used to estimate the average annual percent change (AAPC) over the last ten years. For colorectal cancer, we also computed ASMRs for the 30–39 and 40–49 age group.

We also applied a Poisson joinpoint regression model [13, 14] to the number of deaths in each 5-year age group between 25 and 49, over the period 1990–2023 (or to the most recent year available). Joinpoints were identified iteratively, testing from zero to a maximum of 5 joinpoints. The most recent trend segment identified by the joinpoint model was used to estimate the projected age-specific number of deaths for the year 2026, using a Poisson regression model which included the natural logarithm of the mid-year population as the offset term. Corresponding 95% prediction intervals (PIs) were estimated using Byar’s method [15]. We then computed both age-specific rates and ASMRs, along with their related 95% PIs, using corresponding population data and UN projections.

Statistical analyses were performed using the software R version 4.5.0 (R Core Team (2025), R Foundation for Statistical Computing, Vienna, Austria), SAS version 9.4 (SAS Institute Inc, Cary, NC, USA), and the Joinpoint Regression Program, Version 5.2.0.0 (Statistical Research and Applications Branch, National Cancer Institute; April 2024).

## Results

Table 1 shows the annual average number of deaths, the ASMRs for the triennium 2009–2011 and 2019–2021 and the percent change in rates for all cancers combined and colorectal cancer.

**Table 1:** Age-standardized mortality rates (ASMR) per 100,000 from all cancers combined and colorectal cancer at ages 25–49 years by sex, in 15 upper-middle- and high-income countries and the EU-27 during 2009–2011 and 2019–2021 along with percent change in ASMRs (**Δ**%) and annual average number of deaths during 2019–2021

	Males				Females			
	ASMR 2009–2011	Mean annual number of deaths 2019–2021	ASMR 2019–2021	Δ ASMR %	ASMR 2009–2011	Mean annual number of deaths 2019–2021	ASMR 2019–2021	Δ ASMR %
*All cancers*								
France	36.58	2864	26.77	−26.8	33.89	3082	28.00	−17.4
Germany^a^	26.86	2903	21.05	−21.6	30.41	3517	25.97	−14.6
Italy	27.33	2481	22.72	−16.9	30.44	2939	26.37	−13.4
Netherlands	26.82	647	22.27	−17.0	36.92	804	27.60	−25.2
Poland	37.48	2004	26.82	−28.4	36.56	2105	28.21	−22.8
Spain	30.20	1986	20.49	−32.2	29.76	2221	23.15	−22.2
UK	25.27	2538	22.71	−10.1	31.80	3276	28.76	−9.6
EU-27	32.55	19,797	24.54	−24.6	33.42	21,953	27.31	−18.3
Canada	22.92	1188	18.39	−19.8	29.42	1504	23.03	−21.7
USA	27.51	12,270	21.77	−20.9	31.44	14,930	26.59	−15.4
Argentina	33.61	2148	27.48	−18.2	45.19	3609	46.44	+2.8
Brazil	30.05	10,162	25.67	−14.6	37.57	15,191	36.78	−2.1
Mexico	23.90	5364	25.00	+4.6	35.51	8377	36.26	+2.1
Japan	21.52	3683	16.22	−24.6	26.58	4999	22.43	−15.6
Republic of Korea	33.58	2126	19.44	−42.1	27.44	2247	21.71	−20.9
Australia	25.27	925	20.73	−18.0	28.58	1047	23.00	−19.5
*Colorectal*								
France	2.53	265	2.47	−2.4	2.33	256	2.32	−0.4
Germany^a^	2.70	343	2.48	−8.1	2.20	294	2.16	−1.8
Italy	2.65	299	2.68	+1.1	2.50	237	2.10	−16.0
Netherlands	3.01	93	3.17	+5.3	3.05	82	2.78	−8.9
Poland	3.36	230	3.05	−9.2	2.59	188	2.51	−3.1
Spain	2.79	239	2.40	−14.0	2.40	189	1.97	−17.9
UK	3.15	443	3.94	+25.1	2.56	395	3.46	+35.2
EU-27	2.92	2284	2.80	−4.1	2.52	1925	2.38	−5.6
Canada	2.95	225	3.46	+17.3	2.66	182	2.77	+4.1
USA	3.57	2223	3.93	+10.1	2.78	1672	2.97	+6.8
Argentina	3.67	308	3.91	+6.5	3.24	289	3.72	+14.8
Brazil	2.84	1240	3.11	+9.5	3.08	1310	3.19	+3.6
Mexico	2.17	621	2.91	+34.1	1.69	523	2.26	+33.7
Japan	3.19	695	2.99	−6.3	2.60	561	2.51	−3.5
Republic of Korea	2.92	241	2.19	−25.0	2.41	201	1.91	−20.7
Australia	3.26	163	3.60	+10.4	2.90	150	3.27	+12.8

^a^2019-2020 for Germany

Among males in the EU-27, the ASMR from all cancers combined decreased from 32.55/100,000 in 2009–2011 to 24.54/100,000 in 2019–2021 (−24.6%). In the USA, corresponding figures were 27.51 and 21.77 (−20.9%). All countries considered showed favourable trends, with the exception of Mexico, where mortality increased by 4.6%.

Among females in the EU-27, the ASMR for all cancers combined declined from 33.42/100,000 in 2009–2011 to 27.31/100,000 in 2019–2021 (−18.3%). In the USA, the corresponding figures were 31.44 and 26.59 (−15.4%). Female ASMRs from all cancers decreased for all countries considered, except for Argentina (+ 2.8%) and Mexico (+ 2.1%).

The ASMR from colorectal cancer among males decreased in the EU-27 from 2.92/100,000 in 2009–2011 to 2.80/100,000 in 2019–2021 (−4.1%). In contrast, it increased in the USA from 3.57/100,000 to 3.93/100,000 (+ 10.1%), and also in other countries including Italy (+ 1.1% *vs.* 2009–2011), the Netherlands (+ 5.3%), the UK (+ 25.1%), Canada (+ 17.3%), Argentina (+ 6.5%), Brazil (+ 9.5%), Mexico (+ 34.1%), and Australia (+ 10.4%).

Among females in the EU-27, the ASMR from colorectal cancer decreased from 2.52/100,000 in 2009–2011 to 2.38/100,000 in 2019–2021 (−5.6%). Conversely, it increased in the USA from 2.78 to 2.97 (+ 6.8%), as well as in the UK (+ 35.2%), Canada (+ 4.1%), Argentina (+ 14.8%), Brazil (+ 3.6%), Mexico (+ 33.7%), and Australia (+ 12.8%).

Table 2 reports the analysis for colorectal cancer mortality stratified by age group (30–39 and 40–49 years).

**Table 2:** Age-standardized mortality rates (ASMR) per 100, 000 and annual average number of deaths from colorectal cancer at ages 30–39 and 40–49 years by sex, in 15 upper-middle- and high-income countries and the EU-27 during 2009–2011 and 2019–2021, with the corresponding percent change in rates (Δ%) between the two periods

Age class country	Males				Females			
	ASMR 2009–2011	Mean annual number of deaths 2019–2021	ASMR 2019–2021	Δ ASMR %	ASMR 2009–2011	Mean annual number of deaths 2019–2021	ASMR 2019–2021	Δ ASMR %
*30–39 years*								
France	1.26	62	1.61	+27.8	0.94	56	1.36	+44.7
Germany^a^	1.32	69	1.26	−4.5	1.11	69	1.31	+18.0
Italy	1.27	52	1.50	+18.1	1.18	42	1.23	+4.2
Netherlands	1.45	17	1.57	+8.3	1.53	13	1.19	−22.2
Poland	1.58	41	1.29	−18.4	1.32	41	1.35	+2.3
Spain	1.02	36	1.18	+15.7	1.02	35	1.14	+11.8
UK	1.71	123	2.79	+63.2	1.31	119	2.67	+103.8
EU-27	1.36	417	1.42	+4.4	1.25	395	1.37	+9.6
Canada	1.56	52	1.98	+26.9	1.30	45	1.71	+31.5
USA	1.93	482	2.11	+9.3	1.45	371	1.66	+14.5
Argentina	2.17	82	2.48	+14.3	1.96	82	2.58	+31.6
Brazil	1.64	323	1.86	+13.4	1.83	335	1.92	+4.9
Mexico	1.45	164	1.84	+26.9	1.12	142	1.48	+32.1
Japan	1.59	115	1.62	+1.9	1.39	106	1.55	+11.5
Republic of Korea	1.35	42	1.10	−18.5	1.47	40	1.13	−23.1
Australia	2.01	52	2.81	+39.8	1.46	48	2.57	+76.0
*40–49 years*								
France	5.15	194	4.66	−9.5	4.92	192	4.51	−8.3
Germany^a^	5.69	264	5.08	−10.7	4.54	220	4.30	−5.3
Italy	5.55	239	5.34	−3.8	5.27	192	4.24	−19.5
Netherlands	6.36	73	6.54	+2.8	6.30	68	6.03	−4.3
Poland	7.19	185	6.62	−7.9	5.30	144	5.19	−2.1
Spain	6.17	199	5.07	−17.8	5.15	149	3.85	−25.2
UK	6.21	308	7.39	+19.0	5.07	266	6.26	+23.5
EU-27	6.16	1814	5.77	−6.3	5.19	1491	4.77	−8.1
Canada	5.99	165	6.90	+15.2	5.52	134	5.49	−0.5
USA	7.24	1675	8.00	+10.5	5.68	1257	6.02	+6.0
Argentina	7.02	210	7.37	+5.0	6.13	195	6.87	+12.1
Brazil	5.51	861	6.00	+8.9	5.94	918	6.13	+3.2
Mexico	3.89	424	5.48	+40.9	3.01	353	4.17	+38.5
Japan	6.65	567	6.07	−8.7	5.31	444	4.90	−7.7
Republic of Korea	6.16	190	4.43	−28.1	4.62	157	3.82	−17.3
Australia	6.02	106	6.45	+7.1	5.77	97	5.79	+0.3

^a^2019-2020 for Germany

Among males, the ASMR at ages 30–39 increased by 4.4% in the EU-27 in 2019–2021 compared with 2009–2011, while for the age group 40–49, the ASMR decreased by 6.3% over the same period. The ASMR at ages 30–39 increased in all countries, except Germany, Poland, and the Republic of Korea, with the highest increases observed in the UK (+63.2%), Australia (+39.8%), France (+27.8%), Canada (+26.9%), and Mexico (+26.9%). The ASMR at ages 40–49 increased in six countries including the UK (+19%), Canada (+15.2%), the USA (+10.5%), Argentina (+5%), Brazil (+8.9%), Mexico (+40.9%) and Australia (+7.1%).

Among females, the ASMR at ages 30–39 increased by 9.6% in the EU-27 in 2019–2021 compared with 2009–2011, while for the age group 40–49, the ASMR decreased by 8.1% over the same period. The ASMR at ages 30–39 increased in allcountries, except the Netherlands and the Republic of Korea, with the highest increases observed in the UK (+ 103.8%), Australia (+ 76%) and France (+ 44.7%). The ASMR at ages 40–49 increased in six countries including the UK (+23.5%), the USA (+6%), Argentina (+12.1%), Brazil (+3.2%), Mexico (+38.5%) and Australia (+0.3%).

Table S1 gives mortality rates from lung, pancreatic and breast cancer. As compared to 2009–2011, ASMRs from lung cancer decreased in all selected countries and both sexes. Male lung cancer ASMR in the EU-27 was 3.74/100,000 in 2019–2021 (−43.7% *vs.* 2009–2011), while in the USA it was 2.22/100,000 (−52.3%). ASMRs in 2019–2021 ranged from around 1.2–1.5/100,000 in Mexico and Canada to 5.75/100,000 in France. The largest declines were observed in Poland (−63.2%), Spain (−52.8%), Canada (−56.9%), and the USA (−52.3%). Female ASMR in the EU-27 was 2.34/100,000 in 2019–2021 (−40% *vs.* 2009–2011), while in the USA it was 1.92/100,000 (−55.2%). As in males, the lowest ASMR in 2019–2021 per 100,000 females was in Mexico (0.97) and the highest was in France (3.18). The largest declines among females were observed in the Netherlands (−52.0%), Poland (−50.1%), Canada (−62.5%), and the USA (−55.2%).

Male ASMR per 100,000 from pancreatic cancer in the EU-27 was 1.56 in 2019–2021 (−9.3% *vs.* 2009–2011), while in the USA it was 1.39 (−4.1%). Mortality rates in 2019–2021 varied from 1.03/100,000 in Mexico to 1.73/100,000 in France. Compared to 2009–2011, rates were favourable in most countries, except for France (+0.6%), Brazil (+11.0%), Mexico (+1.0%), and Australia (+7.8%).

Female ASMR from pancreatic cancer in 2019–2021 was 0.95/100,000 in the EU-27 (−5.9% *vs.* 2009–2011) and 0.94/100,000 in the USA (−6.9%). Unfavourable trends were observed in France (+11.1%), Italy (+3.1%), Brazil (+5.3%), and Japan (+8.0%).

In the EU-27, the female breast cancer ASMR in 2019–2021 was 7.90/100,000 (−8.9% *vs.* 2009–2011). The corresponding figure in the USA was 7.04/100,000 (−13.3%). Latin American countries reported the highest rates in 2019–2021, with ASMRs over 9/100,000, and were also the only countries with unfavourable trends compared to 2009–2011.

**Fig. 1:**
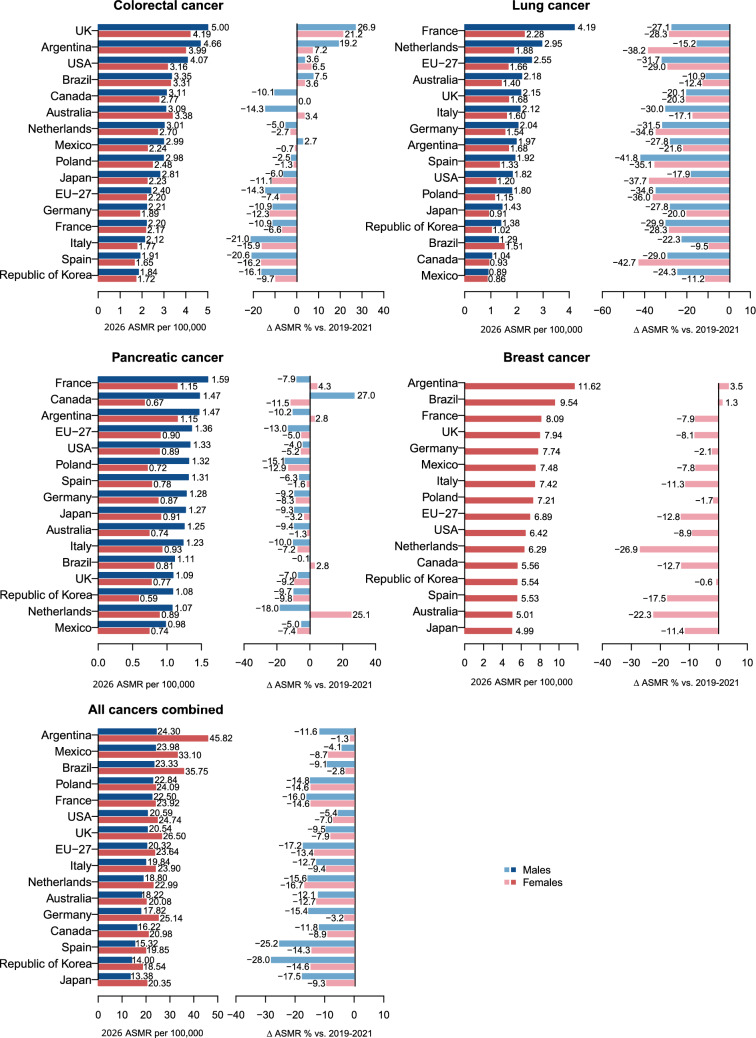
Bar plots reporting the projected age-standardized mortality rates (ASMR) for 2026 from colorectal, lung, pancreatic, and breast cancers and all cancers and corresponding percent changes in rates (Δ%) compared with 2019–2021 among males and females aged 25–49 years in 15 upper-middle and high-income countries and the EU-27

Figure 1 shows the ranking of countries based on the projected ASMRs for 2026 and the corresponding percent change compared to 2019–2021.

Male ASMRs from colorectal cancer in 2026 varied from 5.00/100,000 in the UK to 1.84/100,000 in the Republic of Korea. Projections are unfavourable for the UK (+26.9% *vs.* 2019–2021), Argentina (+19.2%), the USA (+3.6%), Brazil (+7.5%), and Mexico (+2.7%). Among females, projected ASMRs ranged between 1.65/100,000 in Spain to 4.19/100,000 in the UK. Increasing rates were projected in the UK (+21.2% *vs.* 2019–2021), Argentina (+7.2%), the USA (+6.5%), Brazil (+3.6%), and Australia (+3.4%).

Projected ASMRs from lung cancer are expected to be favourable for both sexes and all countries considered. Mexico reported the lowest ASMRs (0.89/100,000 males and 0.86/100,000 females) while France had the highest ones (4.19/100,000 males and 2.28/100,000 females).

Mortality rates from pancreatic cancer for 2026 in males ranged from 0.98/100,000 in Mexico to 1.59/100,000 in France. Projected male trends were favourable in all countries, except Canada (+27.0%). Female projected ASMRs varied between 0.59/100,000 in the Republic of Korea to 1.15/100,000 in France and Argentina. Unfavourable trends were expected in France (+4.3%), Argentina (+2.8%), Brazil (+2.8%), and the Netherlands (+25.1%).

Projected ASMRs from breast cancer ranged between 4.99/100,000 in Japan and 11.62/100,000 in Argentina. Increasing trends were in Argentina (+3.5%) and Brazil (+1.3%). For all other countries, trends were favourable.

Male ASMRs from all cancers combined in 2026 varied from 13.38/100,000 in Japan to 24.30/100,000 in Argentina. Female projected ASMRs ranged between 18.54/100,000 in the Republic of Korea to 45.82/100,000 in Argentina. Compared to 2019–2021, expected ASMRs were favourable for both sexes across all countries.

**Fig. 2:**
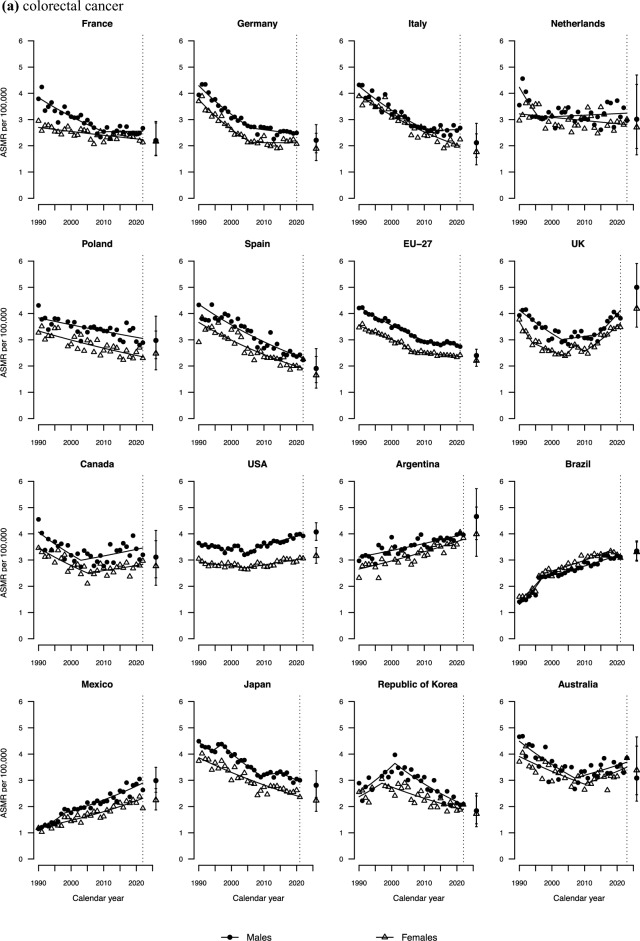

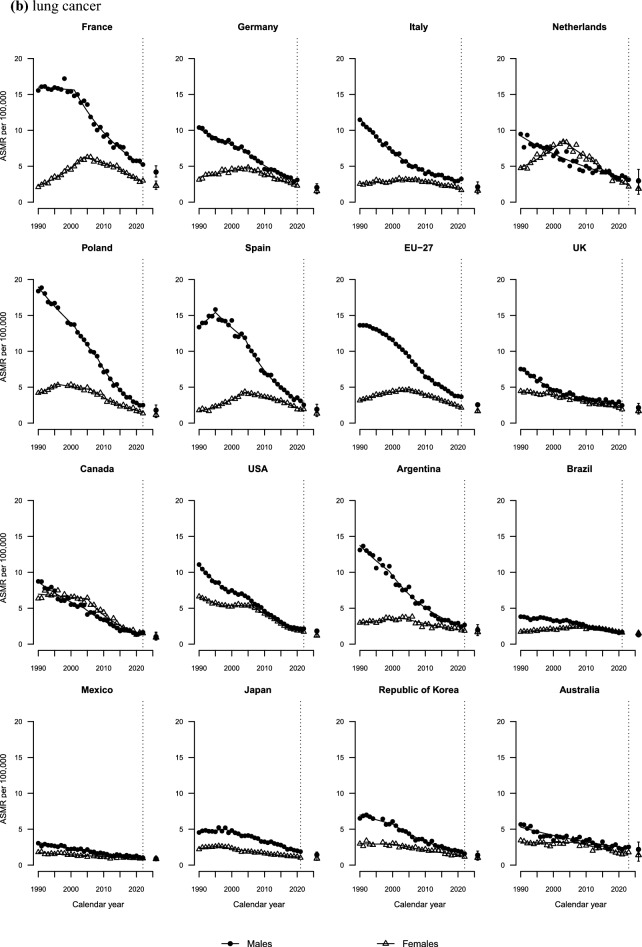

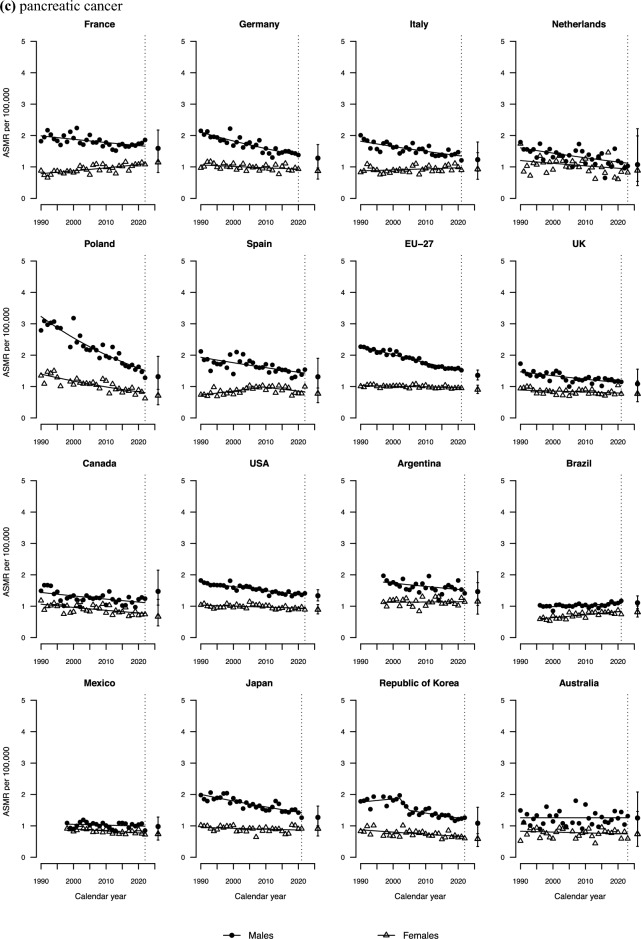

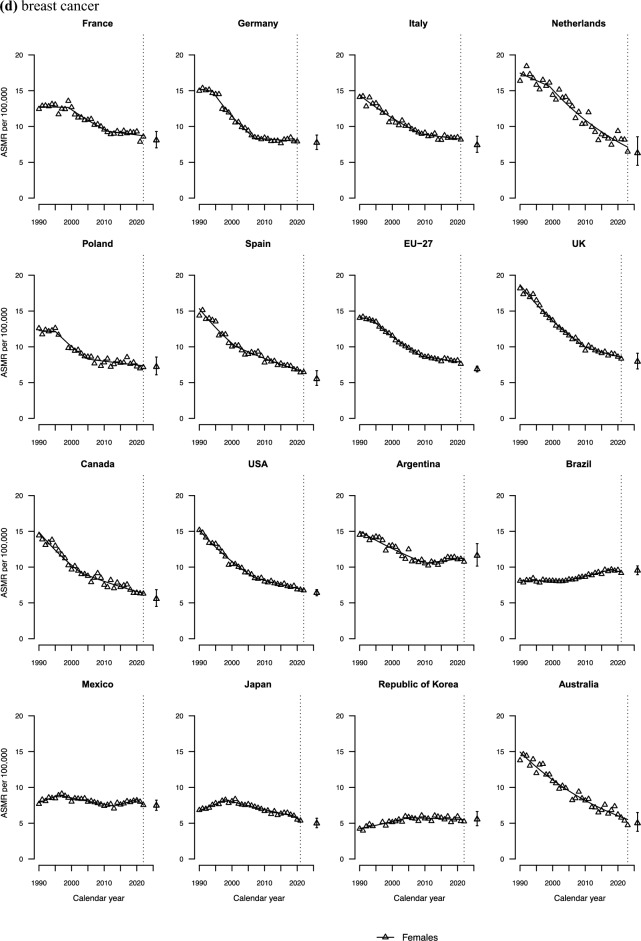

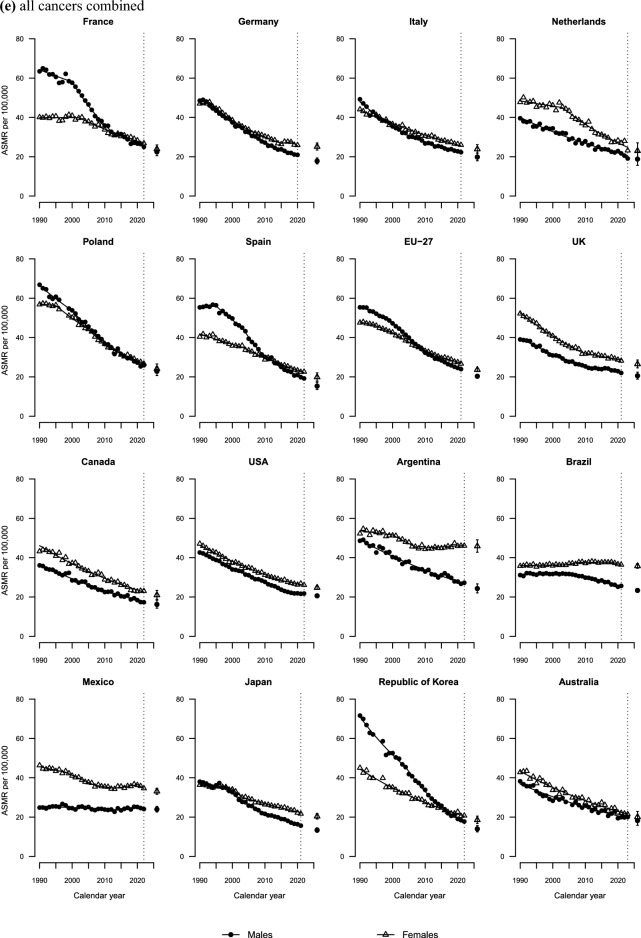
Joinpoint analysis of age-standardized mortality rates (ASMR) from colorectal cancer (**a),** lung cancer (**b),** pancreatic cancer (**c),** breast cancer (**d),** and all cancers combined (**e)** per 100, 000 population aged 25–49 in 15 upper-middle and high-income countries and the EU-27 by sex and projection for the year 2026, along with prediction intervals

Figure 2a shows colorectal cancer mortality trends at age 25–49 over the last three decades, along with the corresponding projected rate for 2026. Unfavourable trends were observed in the UK, Canada, the USA, and Australia, with trends for both sexes showing a marked increase since the mid-2000s. In Mexico, Argentina, and Brazil, ASMRs displayed consistent upward trends throughout the entire study period. An AAPC of over 2% was recorded in both the UK and Mexico over the last decade for both sexes (**Tables S2** and **S3**). In Germany and among Italian males, declines recorded during the 1990s and early 2000s subsequently plateaued. In the Netherlands, mortality rates have remained approximately stable since 1990.

All countries showed decreasing trends in mortality from male lung cancer over the last three decades (Fig. 2b**).** Compared to males, females experienced less pronounced decreases in all countries. In France, Germany, the Netherlands, Spain, the EU-27 and the USA, rates started declining in the mid-2000s, and in Poland in the mid-1990s.

Pancreatic cancer mortality trends between 1990 and 2022 were favourable or stable for both sexes in most countries (Fig. 2c). However, trends increased substantially over the last decade for French females (AAPC of +1.05%) and in Brazil for both sexes (AAPCs of +0.47% in males and +1.33% in females).

Breast cancer mortality rates substantially declined in all countries considered, with the exception of Latin American ones (Fig. 2d). In Argentina, rates have been rising since 2010. In Brazil, mortality rates rose until the late 2010s and subsequently levelled off. Mexico experienced a marked increase in breast cancer mortality during the 2010s, followed by a levelling off.

Overall, cancer mortality declined substantially during the entire period considered, except in Latin America (Fig. 2e). Throughout the study period, rates remained relatively stable among females in Brazil and males in Mexico, while female cancer mortality in Argentina showed a moderate upward trend over the last decade.

## Discussion

Cancer mortality at ages 25–49 is projected to decline in Europe, North America and in upper-middle and high-income countries from other areas of the world, largely driven by reductions in lung and breast cancer mortality. Mortality from pancreatic cancer levelled off after earlier increases. Early onset colorectal cancer mortality is projected to increase substantially in the UK, the USA, most Latin American countries, whereas the trend remained favourable in most European countries, Japan, and the Republic of Korea. When the analysis was restricted to the 30–39 age group, i.e. the generation born in the 1980s, substantial increases in colorectal cancer mortality were observed over the last decade in the UK, North and Latin America, and Australia, and to a lesser extent in most Europe and Japan.

Increases in the incidence of various cancers, including colorectal cancer, have been reported among young adults in several countries [1, 5]. An analysis of changes in colorectal cancer incidence among individuals aged 25–49 and 50–74 in 50 countries between 2008 and 2017 reported upward trends in early-onset colorectal cancer incidence in most countries [16]. This increase was often accompanied by declining rates among adults aged 50–74 years. The growing prevalence of colorectal cancer risk factors among young adults, such as overweight, diabetes, a high intake of processed foods and a sedentary lifestyle, is a possible cause of the global increase in early-onset colorectal cancer [17–19]. A recent European prospective study including over 14,000 early- and later-onset colorectal cancer cases found that adiposity, physical inactivity, smoking, and alcohol consumption are relevant risk factors for early-onset colorectal cancer, with obesity showing a particularly strong association in young males compared to later-onset disease [20]. Moreover, recent colorectal cancer incidence trends in older individuals are favourably influenced by screening and wider adoption of colonoscopy [21]. Screening for colorectal cancer was introduced in the USA in the 1990s. By 2010, around 60% of adults aged 50 to 69 had undergone screening. Individuals born between 1940 and 1965 would have become eligible for screening between 1990 and 2015, and would have progressively benefited from its impact [22]. Similar figures were also reported from Europe [23, 24], partly explaining the difference in mortality patterns between young and older age groups.

Lung cancer mortality is expected to decrease further by 2026 in almost all countries, with a greater decline among males. This pattern likely reflects historical smoking prevalence and cessation trends across successive generations, as smoking prevalence peaked later among females than among males [1, 25].

Mortality rates from pancreatic cancer are projected to level off. US Cancer Statistics data indicated that a rising incidence of pancreatic cancer among US adults aged 15–39 years [26]. However, mortality rates were stable in the corresponding age group. This may be partly explained by increased detection of small, early-stage pancreatic tumors—particularly neuroendocrine tumors—rather than a true increase in pancreatic adenocarcinoma. Such trends in incidence likely reflect earlier or incidental diagnoses driven by more widespread use of imaging and improved detection, rather than an actual increase in disease occurrence.

In 2026, breast cancer mortality is projected to be favourable, with a few exceptions, mainly in Latin America. Improved diagnosis and treatment had a key role in the favourable trends of breast cancer [27]. For Latin American countries, the increase in mortality is likely due to delayed diagnosis and limited access to innovative treatments [28].

Our study provides a comprehensive and up-to-date assessment of cancer mortality patterns and temporal trends among young adults. Our analyses are based on the most recent and reliable official mortality data from the WHO database. To ensure validity of our findings, analyses were restricted to major countries with high-quality and high-coverage mortality data. However, the projections should be interpreted with caution, as they rely on the assumption that no major changes will occur in the factors underlying recent mortality trends. In addition, the projection approach, based on age-specific joinpoint models, may be less sensitive to substantial cohort effects. Finally, the relatively small number of deaths in younger age groups limited the precision in estimating temporal changes in mortality rates.

In conclusion, overall mortality trends reflected the main types of cancer, i.e. lung cancer in males and breast cancer in females. These trends likely reflect the impact of tobacco control measures as well as earlier diagnosis and improved treatments for breast cancer. Conversely, colorectal cancer mortality is projected to increase in several countries, particularly among individuals younger than 40 years, although it contributes only marginally to overall cancer mortality projections. This pattern is related to the growing prevalence of overweight, obesity, diabetes, and other metabolic disorders in younger populations.

## Supplementary Information

Below is the link to the electronic supplementary material.

Supplementary file1 (DOCX 84 KB)

## Data Availability

Data derived from a source in the public domain. We retrieved death counts from the World Health Organization (WHO) database and resident population data from the United Nations World Population Prospects database.
