# Supplementary material for: Cancer mortality projections through 2026 among young adults in 15 upper-middle and high-income countries with focus on colorectal cancer
Source: Cancer Causes Control. 2026 Apr 3;37(5):79. doi: 10.1007/s10552-026-02149-w (PMC13048963; doi:10.1007/s10552-026-02149-w)
Supplement: Supplementary file 1 — Supplementary file1 (DOCX 84 KB) [file 10552_2026_2149_MOESM1_ESM.docx]

**SUPPLEMENTARY MATERIAL**

**Manuscript title:** Cancer mortality projections through 2026 among young adults in 15 upper-middle and high-income countries with focus on colorectal cancer

**Authors:** Silvia Mignozzi, Claudia Santucci, Gianfranco Alicandro, Margherita Pizzato, Prabhat Jha, Eva Negri, Carlo La Vecchia

**Corresponding to:** Silvia Mignozzi, MSc (silvia.mignozzi@unimi.it)

**Table of contents**

[**Supplementary Table 1.** Annual average number of deaths and age-standardized mortality rates (ASMR) per 100,000 from lung, pancreatic and breast cancer 25-49 years by sex, in 15 upper-middle- and high-income countries and EU-27 during 2009-2011 and 2019-2021 along with percent change in ASMRs (Δ%). 2](#_Toc221790564)

[**Supplementary Table 2.** Joinpoint analysis from 1990 to the most available year for males aged 25-49 years, by country and cancer site. 3](#_Toc221790565)

[**Supplementary Table 3.** Joinpoint analysis from 1990 to the most available year for females aged 25-49 years, by country and cancer site. 5](#_Toc221790566)

# **Supplementary Table 1.** Annual average number of deaths and age-standardized mortality rates (ASMR) per 100,000 from lung, pancreatic and breast cancer 25-49 years by sex, in 15 upper-middle- and high-income countries and EU-27 during 2009-2011 and 2019-2021 along with percent change in ASMRs (Δ%).

| **Cancer site**  **Country** | **Males** | | | |  | **Females** | | | |
| --- | --- | --- | --- | --- | --- | --- | --- | --- | --- |
|  | **ASMR 2009-2011** | **Mean number of deaths**  **2019-2021** | **ASMR 2019-2021** | **Δ**  **ASMR %** |  | **ASMR 2009-2011** | **Mean number of deaths**  **2019-2021** | **ASMR 2019-2021** | **Δ**  **ASMR %** |
| **Lung** |  |  |  |  |  |  |  |  |  |
| France | 9.66 | 634 | 5.75 | -40.5 |  | 5.29 | 359 | 3.18 | -39.9 |
| Germany ^a^ | 4.86 | 413 | 2.98 | -38.7 |  | 3.84 | 323 | 2.35 | -38.8 |
| Italy | 4.23 | 350 | 3.03 | -28.4 |  | 2.80 | 226 | 1.93 | -31.1 |
| Netherlands | 4.67 | 103 | 3.48 | -25.5 |  | 6.33 | 91 | 3.04 | -52.0 |
| Poland | 7.47 | 206 | 2.75 | -63.2 |  | 3.59 | 133 | 1.79 | -50.1 |
| Spain | 6.99 | 335 | 3.30 | -52.8 |  | 3.65 | 205 | 2.05 | -43.8 |
| UK | 3.39 | 302 | 2.69 | -20.6 |  | 2.85 | 242 | 2.11 | -26.0 |
| EU-27 | 6.64 | 3100 | 3.74 | -43.7 |  | 3.90 | 1923 | 2.34 | -40.0 |
| Canada | 3.39 | 94 | 1.46 | -56.9 |  | 4.32 | 106 | 1.62 | -62.5 |
| USA | 4.65 | 1247 | 2.22 | -52.3 |  | 4.29 | 1075 | 1.92 | -55.2 |
| Argentina | 5.34 | 209 | 2.73 | -48.9 |  | 2.63 | 164 | 2.14 | -18.6 |
| Brazil | 2.52 | 652 | 1.66 | -34.1 |  | 2.25 | 678 | 1.67 | -25.8 |
| Mexico | 1.61 | 252 | 1.18 | -26.7 |  | 1.13 | 222 | 0.97 | -14.2 |
| Japan | 3.22 | 477 | 1.98 | -38.5 |  | 1.66 | 264 | 1.14 | -31.3 |
| Republic of Korea | 3.28 | 221 | 1.97 | -39.9 |  | 2.10 | 151 | 1.43 | -31.9 |
| Australia | 3.42 | 109 | 2.45 | -28.4 |  | 2.95 | 73 | 1.60 | -45.8 |
| **Pancreatic** |  |  |  |  |  |  |  |  |  |
| France | 1.72 | 189 | 1.73 | +0.6 |  | 0.99 | 124 | 1.10 | +11.1 |
| Germany ^a^ | 1.49 | 197 | 1.41 | -5.4 |  | 0.98 | 131 | 0.95 | -3.1 |
| Italy | 1.58 | 157 | 1.37 | -13.3 |  | 0.97 | 115 | 1.00 | +3.1 |
| Netherlands | 1.33 | 39 | 1.31 | -1.5 |  | 1.11 | 21 | 0.71 | -36.0 |
| Poland | 2.08 | 116 | 1.55 | -25.5 |  | 1.07 | 61 | 0.82 | -23.4 |
| Spain | 1.59 | 143 | 1.40 | -11.9 |  | 0.97 | 79 | 0.79 | -18.6 |
| UK | 1.25 | 130 | 1.17 | -6.4 |  | 0.85 | 98 | 0.85 | 0.0 |
| EU-27 | 1.72 | 1288 | 1.56 | -9.3 |  | 1.01 | 783 | 0.95 | -5.9 |
| Canada | 1.28 | 74 | 1.16 | -9.4 |  | 0.97 | 49 | 0.76 | -21.6 |
| USA | 1.45 | 784 | 1.39 | -4.1 |  | 1.01 | 527 | 0.94 | -6.9 |
| Argentina | 1.76 | 125 | 1.63 | -7.4 |  | 1.14 | 86 | 1.12 | -1.8 |
| Brazil | 1.00 | 437 | 1.11 | +11.0 |  | 0.75 | 320 | 0.79 | +5.3 |
| Mexico | 1.02 | 218 | 1.03 | +1.0 |  | 0.83 | 183 | 0.80 | -3.6 |
| Japan | 1.51 | 335 | 1.40 | -7.3 |  | 0.87 | 218 | 0.94 | +8.0 |
| Republic of Korea | 1.51 | 135 | 1.20 | -20.5 |  | 0.75 | 70 | 0.65 | -13.3 |
| Australia | 1.28 | 61 | 1.38 | 7.8 |  | 0.83 | 34 | 0.75 | -9.6 |
| **Breast** |  |  |  |  |  |  |  |  |  |
| France |  |  |  |  |  | 9.60 | 975 | 8.78 | -8.5 |
| Germany ^a^ |  |  |  |  |  | 8.30 | 1075 | 7.91 | -4.7 |
| Italy |  |  |  |  |  | 8.91 | 948 | 8.36 | -6.2 |
| Netherlands |  |  |  |  |  | 10.91 | 251 | 8.61 | -21.1 |
| Poland |  |  |  |  |  | 7.82 | 557 | 7.34 | -6.1 |
| Spain |  |  |  |  |  | 8.28 | 659 | 6.70 | -19.1 |
| UK |  |  |  |  |  | 9.96 | 989 | 8.64 | -13.3 |
| EU-27 |  |  |  |  |  | 8.67 | 6414 | 7.90 | -8.9 |
| Canada |  |  |  |  |  | 7.73 | 419 | 6.37 | -17.6 |
| USA |  |  |  |  |  | 8.12 | 3970 | 7.04 | -13.3 |
| Argentina |  |  |  |  |  | 10.58 | 870 | 11.22 | +6.0 |
| Brazil |  |  |  |  |  | 8.75 | 3918 | 9.42 | +7.7 |
| Mexico |  |  |  |  |  | 7.53 | 1871 | 8.11 | +7.7 |
| Japan |  |  |  |  |  | 6.81 | 1285 | 5.63 | -17.3 |
| Republic of Korea |  |  |  |  |  | 5.80 | 587 | 5.58 | -3.8 |
| Australia |  |  |  |  |  | 8.34 | 294 | 6.45 | -22.7 |

^a^ 2020 for Germany.

# **Supplementary Table 2.** Joinpoint analysis from 1990 to the most available year for males aged 25-49 years, by country and cancer site.

| **Cancer site**  **Country** | **Year 1** | **APC 1** | **Year 2** | **APC 2** | **Year 3** | **APC 3** | **Year 4** | **APC 4** | **Year 5** | **APC 5** | **Year 6** | **APC 6** | **AAPC^a^** |
| --- | --- | --- | --- | --- | --- | --- | --- | --- | --- | --- | --- | --- | --- |
|  |  |  |  |  |  |  |  |  |  |  |  |  |  |
| **Colorectum** |  |  |  |  |  |  |  |  |  |  |  |  |  |
| France | 1990-2010 | -2.05* | 2010-2022 | +0.08 |  |  |  |  |  |  |  |  | +0.08 |
| Germany | 1990-2006 | -2.91* | 2006-2020 | -0.62 |  |  |  |  |  |  |  |  | -0.62 |
| Italy | 1990-2011 | -2.38* | 2011-2021 | +0.18 |  |  |  |  |  |  |  |  | +0.18 |
| Netherlands | 1990-1996 | -5.13* | 1996-2023 | +0.19 |  |  |  |  |  |  |  |  | +0.19 |
| Poland | 1990-2022 | -0.71* |  |  |  |  |  |  |  |  |  |  | -0.71* |
| Spain | 1990-2022 | -1.97* |  |  |  |  |  |  |  |  |  |  | -1.97* |
| UK | 1990-2003 | -2.42* | 2003-2014 | +0.57 | 2014-2021 | +3.68* |  |  |  |  |  |  | +2.98* |
| EU-27 | 1990-2011 | -1.79* | 2011-2021 | -0.38 |  |  |  |  |  |  |  |  | -0.38 |
| Canada | 1990-2003 | -2.33* | 2003-2022 | +0.77* |  |  |  |  |  |  |  |  | +0.77* |
| USA | 1990-1998 | -0.96* | 1998-2002 | +1.08 | 2002-2005 | -3.38 | 2005-2008 | +3.36 | 2008-2022 | +0.89* |  |  | +0.89* |
| Argentina | 1990-2022 | +0.77* |  |  |  |  |  |  |  |  |  |  | +0.77* |
| Brazil | 1990-1997 | +8.58* | 1997-2021 | +1.37* |  |  |  |  |  |  |  |  | +1.37* |
| Mexico | 1990-1998 | +5.94* | 1998-2022 | +2.12* |  |  |  |  |  |  |  |  | +2.12* |
| Japan | 1990-1994 | -2.02* | 1994-1997 | +2.05 | 1997-2009 | -2.61* | 2009-2015 | +0.59 | 2015-2021 | -1.80* |  |  | -1.01* |
| Republic of Korea | 1990-2001 | +3.32* | 2001-2022 | -2.66* |  |  |  |  |  |  |  |  | -2.66* |
| Australia | 1990-2007 | -2.18* | 2007-2023 | +1.12* |  |  |  |  |  |  |  |  | +1.12* |
| **Lung** |  |  |  |  |  |  |  |  |  |  |  |  |  |
| France | 1990-2001 | -0.22 | 2001-2022 | -5.16* |  |  |  |  |  |  |  |  | -5.16* |
| Germany | 1990-2003 | -2.36* | 2003-2020 | -5.35* |  |  |  |  |  |  |  |  | -5.35* |
| Italy | 1990-2010 | -5.01* | 2010-2021 | -3.08* |  |  |  |  |  |  |  |  | -3.08* |
| Netherlands | 1990-2023 | -3.12* |  |  |  |  |  |  |  |  |  |  | -3.12* |
| Poland | 1990-2001 | -3.02* | 2001-2008 | -5.44* | 2008-2022 | -9.33* |  |  |  |  |  |  | -9.33* |
| Spain | 1990-1995 | 2.77* | 1995-2004 | -2.94* | 2004-2022 | -7.67* |  |  |  |  |  |  | -7.67* |
| UK | 1990-2003 | -4.81* | 2003-2021 | -2.34* |  |  |  |  |  |  |  |  | -2.34* |
| EU-27 | 1990-1997 | -1.18* | 1997-2004 | -3.50* | 2004-2010 | -6.56* | 2010-2016 | -5.17* | 2016-2019 | -7.36* | 2019-2021 | -1.75 | -5.16* |
| Canada | 1990-2004 | -3.83* | 2004-2022 | -7.03* |  |  |  |  |  |  |  |  | -7.03* |
| USA | 1990-1998 | -4.48* | 1998-2004 | -1.70* | 2004-2012 | -6.24* | 2012-2018 | -8.88* | 2018-2022 | -2.36 |  |  | -6.04* |
| Argentina | 1990-1999 | -3.32* | 1999-2022 | -6.13* |  |  |  |  |  |  |  |  | -6.13* |
| Brazil | 1990-2005 | -1.20* | 2005-2021 | -4.2* |  |  |  |  |  |  |  |  | -4.20* |
| Mexico | 1990-2022 | -3.30* |  |  |  |  |  |  |  |  |  |  | -3.30* |
| Japan | 1990-1998 | +0.95 | 1998-2013 | -3.23* | 2013-2021 | -6.03* |  |  |  |  |  |  | -5.73* |
| Republic of Korea | 1990-2000 | -1.99* | 2000-2022 | -5.36* |  |  |  |  |  |  |  |  | -5.36* |
| Australia | 1990-2023 | -2.44* |  |  |  |  |  |  |  |  |  |  | -2.44* |
| **Pancreas** |  |  |  |  |  |  |  |  |  |  |  |  |  |
| France | 1990-2022 | -0.56* |  |  |  |  |  |  |  |  |  |  | -0.56* |
| Germany | 1990-2020 | -1.42* |  |  |  |  |  |  |  |  |  |  | -1.42* |
| Italy | 1990-2021 | -0.97* |  |  |  |  |  |  |  |  |  |  | -0.97* |
| Netherlands | 1990-2023 | -1.09* |  |  |  |  |  |  |  |  |  |  | -1.09* |
| Poland | 1990-2022 | -2.34* |  |  |  |  |  |  |  |  |  |  | -2.34* |
| Spain | 1990-2022 | -0.94* |  |  |  |  |  |  |  |  |  |  | -0.94* |
| UK | 1990-2021 | -0.87* |  |  |  |  |  |  |  |  |  |  | -0.87* |
| EU-27 | 1990-2021 | -1.36* |  |  |  |  |  |  |  |  |  |  | -1.36* |
| Canada | 1990-2022 | -0.80* |  |  |  |  |  |  |  |  |  |  | -0.80* |
| USA | 1990-2022 | -0.84* |  |  |  |  |  |  |  |  |  |  | -0.84* |
| Argentina | 1997-2022 | -0.62* |  |  |  |  |  |  |  |  |  |  | -0.62* |
| Brazil | 1996-2021 | +0.47* |  |  |  |  |  |  |  |  |  |  | +0.47* |
| Mexico | 1998-2022 | -0.26 |  |  |  |  |  |  |  |  |  |  | -0.26 |
| Japan | 1990-2021 | -1.10* |  |  |  |  |  |  |  |  |  |  | -1.10* |
| Republic of Korea | 1990-2002 | +0.57 | 2002-2005 | -7.41 | 2005-2022 | -1.18* |  |  |  |  |  |  | -1.18* |
| Australia | 1990-2023 | 0 |  |  |  |  |  |  |  |  |  |  | 0.00 |
| **All cancers** |  |  |  |  |  |  |  |  |  |  |  |  |  |
| France | 1990-2000 | -0.99* | 2000-2012 | -4.61* | 2012-2022 | -2.67* |  |  |  |  |  |  | -2.67* |
| Germany | 1990-1992 | -1.16 | 1992-2020 | -3.03* |  |  |  |  |  |  |  |  | -3.03* |
| Italy | 1990-2005 | -2.95* | 2005-2021 | -2.01* |  |  |  |  |  |  |  |  | -2.01* |
| Netherlands | 1990-2023 | -1.99* |  |  |  |  |  |  |  |  |  |  | -1.99* |
| Poland | 1990-2000 | -2.12* | 2000-2022 | -3.41* |  |  |  |  |  |  |  |  | -3.41* |
| Spain | 1990-1995 | +0.17 | 1995-2003 | -2.67* | 2003-2009 | -5.61* | 2009-2022 | -3.89* |  |  |  |  | -3.89* |
| UK | 1990-2011 | -2.29* | 2011-2017 | -0.22 | 2017-2021 | -2.07* |  |  |  |  |  |  | -1.05* |
| EU-27 | 1990-1998 | -1.43* | 1998-2003 | -2.88* | 2003-2011 | -3.92* | 2011-2021 | -2.68* |  |  |  |  | -2.68* |
| Canada | 1990-2022 | -2.29* |  |  |  |  |  |  |  |  |  |  | -2.29* |
| USA | 1990-2009 | -2.20* | 2009-2018 | -2.78* | 2018-2022 | -0.30 |  |  |  |  |  |  | -1.69* |
| Argentina | 1990-2022 | -1.87* |  |  |  |  |  |  |  |  |  |  | -1.87* |
| Brazil | 1990-2006 | +0.05 | 2006-2021 | -1.46* |  |  |  |  |  |  |  |  | -1.46* |
| Mexico | 1990-2013 | -0.33* | 2013-2022 | +0.51 |  |  |  |  |  |  |  |  | +0.51 |
| Japan | 1990-1998 | -0.94* | 1998-2008 | -4.03* | 2008-2021 | -2.83* |  |  |  |  |  |  | -2.83* |
| Republic of Korea | 1990-2004 | -3.12* | 2004-2022 | -5.21* |  |  |  |  |  |  |  |  | -5.21* |
| Australia | 1990-2023 | -1.91* |  |  |  |  |  |  |  |  |  |  | -1.91* |

AAPC, average annual percent change. ^a^ AAPC computed over the last 10 years.

# **Supplementary Table 3.** Joinpoint analysis from 1990 to the most available year for females aged 25-49 years, by country and cancer site.

| **Cancer site**  **Country** | **Year 1** | **APC 1** | **Year 2** | **APC 2** | **Year 3** | **APC 3** | **Year 4** | **APC 4** | **Year 5** | **APC 5** | **AAPC^a^** |
| --- | --- | --- | --- | --- | --- | --- | --- | --- | --- | --- | --- |
|  |  |  |  |  |  |  |  |  |  |  |  |
| **Colorectum** |  |  |  |  |  |  |  |  |  |  |  |
| France | 1990-2022 | -0.59* |  |  |  |  |  |  |  |  | -0.59* |
| Germany | 1990-2006 | -3.51* | 2006-2020 | -0.03 |  |  |  |  |  |  | -0.03 |
| Italy | 1990-2021 | -2.18* |  |  |  |  |  |  |  |  | -2.18* |
| Netherlands | 1990-2023 | -0.41* |  |  |  |  |  |  |  |  | -0.41* |
| Poland | 1990-2022 | -1.09* |  |  |  |  |  |  |  |  | -1.09* |
| Spain | 1990-2022 | -2.01* |  |  |  |  |  |  |  |  | -2.01* |
| UK | 1990-1994 | -5.95* | 1994-2004 | -1.97* | 2004-2007 | +5.92 | 2007-2010 | -3.18 | 2010-2021 | +3.27* | +3.27* |
| EU-27 | 1990-2003 | -1.62* | 2003-2006 | -3.83 | 2006-2021 | -0.50* |  |  |  |  | -0.50* |
| Canada | 1990-2005 | -2.13* | 2005-2022 | +0.72* |  |  |  |  |  |  | +0.72* |
| USA | 1990-2004 | -0.58* | 2004-2022 | +0.70* |  |  |  |  |  |  | +0.70* |
| Argentina | 1990-2022 | +1.11* |  |  |  |  |  |  |  |  | +1.11* |
| Brazil | 1990-1998 | +6.93* | 1998-2018 | +1.47* | 2018-2021 | -2.65 |  |  |  |  | +0.07 |
| Mexico | 1990-2022 | +2.08* |  |  |  |  |  |  |  |  | +2.08* |
| Japan | 1990-2021 | -1.56* |  |  |  |  |  |  |  |  | -1.56* |
| Republic of Korea | 1990-1997 | +2.85 | 1997-2022 | -1.70* |  |  |  |  |  |  | -1.70* |
| Australia | 1990-2010 | -1.56* | 2010-2023 | +1.58* |  |  |  |  |  |  | +1.58* |
| **Lung** |  |  |  |  |  |  |  |  |  |  |  |
| France | 1990-2004 | +7.50* | 2004-2014 | -2.59* | 2014-2022 | -6.58* |  |  |  |  | -6.15* |
| Germany | 1990-2005 | +2.29* | 2005-2020 | -4.58* |  |  |  |  |  |  | -4.58* |
| Italy | 1990-2007 | +1.32* | 2007-2021 | -3.70* |  |  |  |  |  |  | -3.70* |
| Netherlands | 1990-2003 | +4.55* | 2003-2012 | -4.25* | 2012-2023 | -7.98* |  |  |  |  | -7.98* |
| Poland | 1990-1996 | +4.63* | 1996-2006 | -1.42 | 2006-2022 | -6.81* |  |  |  |  | -6.81* |
| Spain | 1990-2004 | +7.12* | 2004-2015 | -2.83* | 2015-2022 | -7.65* |  |  |  |  | -6.60* |
| UK | 1990-1999 | -0.88 | 1999-2021 | -2.84* |  |  |  |  |  |  | -2.84* |
| EU-27 | 1990-1997 | +3.94* | 1997-2005 | +1.53* | 2005-2014 | -3.54* | 2014-2021 | -6.04* |  |  | -5.49* |
| Canada | 1990-2005 | -0.65 | 2005-2022 | -8.54* |  |  |  |  |  |  | -8.54* |
| USA | 1990-1997 | -2.90* | 1997-2006 | 0 | 2006-2012 | -5.58* | 2012-2018 | -9.26* | 2018-2022 | -4.98* | -7.38* |
| Argentina | 1990-2003 | +1.51* | 2003-2022 | -3.37* |  |  |  |  |  |  | -3.37* |
| Brazil | 1990-2008 | +2.02* | 2008-2021 | -2.81* |  |  |  |  |  |  | -2.81* |
| Mexico | 1990-2022 | -1.94* |  |  |  |  |  |  |  |  | -1.94* |
| Japan | 1990-1996 | +2.31* | 1996-2021 | -3.48* |  |  |  |  |  |  | -3.48* |
| Republic of Korea | 1990-2001 | -0.31 | 2001-2022 | -3.62* |  |  |  |  |  |  | -3.62* |
| Australia | 1990-2007 | +0.25 | 2007-2023 | -4.24* |  |  |  |  |  |  | -4.24* |
| **Pancreas** |  |  |  |  |  |  |  |  |  |  |  |
| France | 1990-2022 | +1.05* |  |  |  |  |  |  |  |  | +1.05* |
| Germany | 1990-2020 | -0.44* |  |  |  |  |  |  |  |  | -0.44* |
| Italy | 1990-2021 | +0.36 |  |  |  |  |  |  |  |  | +0.36 |
| Netherlands | 1990-2023 | -0.75 |  |  |  |  |  |  |  |  | -0.75 |
| Poland | 1990-2022 | -1.71* |  |  |  |  |  |  |  |  | -1.71* |
| Spain | 1990-2009 | +1.60* | 2009-2022 | -1.30 |  |  |  |  |  |  | -1.30 |
| UK | 1990-2021 | -0.37 |  |  |  |  |  |  |  |  | -0.37 |
| EU-27 | 1990-2021 | -0.23* |  |  |  |  |  |  |  |  | -0.23* |
| Canada | 1990-2022 | -1.01* |  |  |  |  |  |  |  |  | -1.01* |
| USA | 1990-2022 | -0.32* |  |  |  |  |  |  |  |  | -0.32* |
| Argentina | 1997-2022 | +0.07 |  |  |  |  |  |  |  |  | +0.07 |
| Brazil | 1996-2021 | +1.33* |  |  |  |  |  |  |  |  | +1.33* |
| Mexico | 1998-2022 | -0.75* |  |  |  |  |  |  |  |  | -0.75* |
| Japan | 1990-2021 | -0.30 |  |  |  |  |  |  |  |  | -0.30 |
| Republic of Korea | 1990-2022 | -0.88* |  |  |  |  |  |  |  |  | -0.88* |
| Australia | 1990-2023 | -0.33 |  |  |  |  |  |  |  |  | -0.33 |
| **Breast** |  |  |  |  |  |  |  |  |  |  |  |
| France | 1990-1999 | -0.08 | 1999-2011 | -2.50* | 2011-2022 | -0.63 |  |  |  |  | -0.63 |
| Germany | 1990-1994 | -0.42 | 1994-2007 | -4.36* | 2007-2020 | -0.40 |  |  |  |  | -0.40 |
| Italy | 1990-2009 | -2.48* | 2009-2021 | -0.66* |  |  |  |  |  |  | -0.66* |
| Netherlands | 1990-1999 | -1.22 | 1999-2023 | -3.22* |  |  |  |  |  |  | -3.22* |
| Poland | 1990-1995 | -0.18 | 1995-2005 | -3.77* | 2005-2022 | -0.69* |  |  |  |  | -0.69* |
| Spain | 1990-2001 | -3.73* | 2001-2022 | -2.06* |  |  |  |  |  |  | -2.06* |
| UK | 1990-2010 | -3.10* | 2010-2021 | -1.40* |  |  |  |  |  |  | -1.40* |
| EU-27 | 1990-1994 | -0.80 | 1994-2009 | -2.99* | 2009-2021 | -0.85* |  |  |  |  | -0.85* |
| Canada | 1990-2003 | -3.66* | 2003-2022 | -1.93* |  |  |  |  |  |  | -1.93* |
| USA | 1990-2007 | -3.35* | 2007-2022 | -1.45* |  |  |  |  |  |  | -1.45* |
| Argentina | 1990-2010 | -1.75* | 2010-2022 | +0.54 |  |  |  |  |  |  | +0.54 |
| Brazil | 1990-2004 | -0.02 | 2004-2018 | +1.30* | 2018-2021 | -1.37 |  |  |  |  | +0.40 |
| Mexico | 1990-1996 | +2.12* | 1996-2013 | -1.10* | 2013-2020 | +1.62* | 2020-2022 | -4.42 |  |  | +0.25 |
| Japan | 1990-1998 | +2.53* | 1998-2019 | -1.51* | 2019-2021 | -6.16 |  |  |  |  | -2.57* |
| Republic of Korea | 1990-2005 | +2.08* | 2005-2022 | -0.29 |  |  |  |  |  |  | -0.29 |
| Australia | 1990-2023 | -2.98* |  |  |  |  |  |  |  |  | -2.98* |
| **All cancers** |  |  |  |  |  |  |  |  |  |  |  |
| France | 1990-2002 | -0.02 | 2002-2022 | -1.96* |  |  |  |  |  |  | -1.96* |
| Germany | 1990-1993 | +0.43 | 1993-2001 | -3.12* | 2001-2015 | -2.27* | 2015-2018 | +1.00 | 2018-2020 | -3.71 | -1.52 |
| Italy | 1990-2021 | -1.71* |  |  |  |  |  |  |  |  | -1.71* |
| Netherlands | 1990-2003 | -0.56* | 2003-2023 | -2.94* |  |  |  |  |  |  | -2.94* |
| Poland | 1990-1995 | -0.34 | 1995-2005 | -2.40* | 2005-2011 | -3.75* | 2011-2022 | -2.38* |  |  | -2.38* |
| Spain | 1990-2003 | -1.35* | 2003-2022 | -2.37* |  |  |  |  |  |  | -2.37* |
| UK | 1990-2009 | -2.52* | 2009-2021 | -1.01* |  |  |  |  |  |  | -1.01* |
| EU-27 | 1990-1994 | -0.67 | 1994-2000 | -1.54* | 2000-2013 | -2.36* | 2013-2021 | -1.77* |  |  | -1.83* |
| Canada | 1990-2022 | -2.21* |  |  |  |  |  |  |  |  | -2.21* |
| USA | 1990-2015 | -1.95* | 2015-2022 | -1.26* |  |  |  |  |  |  | -1.41* |
| Argentina | 1990-2000 | -0.40 | 2000-2008 | -1.77* | 2008-2022 | +0.23* |  |  |  |  | +0.23* |
| Brazil | 1990-2004 | +0.08 | 2004-2008 | +0.84 | 2008-2018 | +0.02 | 2018-2021 | -1.22* |  |  | -0.39* |
| Mexico | 1990-1998 | -0.78* | 1998-2007 | -1.85* | 2007-2013 | -0.73 | 2013-2020 | +0.76* | 2020-2022 | -2.55 | +0.02 |
| Japan | 1990-2000 | -0.62* | 2000-2003 | -4.25* | 2003-2021 | -1.65* |  |  |  |  | -1.65* |
| Republic of Korea | 1990-2022 | -2.39* |  |  |  |  |  |  |  |  | -2.39* |
| Australia | 1990-2023 | -2.10* |  |  |  |  |  |  |  |  | -2.10* |

AAPC, average annual percent change. ^a^ AAPC computed over the last 10 years.
